# Syrup of Wild Cherry Bark

**Published:** 1842-05-14

**Authors:** 


### ANALECTA.

*Syrup of Wild Cherry Bark.*—This is an elegant preparation, much used in the practice of Philadelphia, which is in some respects superior to the cold infusion made by maceration, as it contains the virtues of the article in a more concentrated form, and is capable of preservation for a long time. Messrs. Procter and Turnpenny, in the April number of the Journal of Pharmacy, give the following formula for preparing it:—

Take of Wild Cherry bark, in powder, ℥iv. Water, ℥xii. Sugar, in coarse powder, ℥xxiv.

Macerate the bark in the water for forty-eight hours; put the mixture into a displacement apparatus; return the fluid that passes several times, until it becomes transparent, and then add sufficient water to displace twelve fluid ounces of infusion. Place the sugar in a displacement funnel, and pass and repass the infusion through it, until it is all dissolved. Lastly, preserve in well stopped bottles.

